# Evolving trends in neuropsychological profiles of post COVID-19 condition: A 1-year follow-up in individuals with cognitive complaints

**DOI:** 10.1371/journal.pone.0302415

**Published:** 2024-08-08

**Authors:** Nicholas Grunden, Marco Calabria, Carmen García-Sánchez, Catalina Pons, Juan Antonio Arroyo, Beatriz Gómez-Ansón, Marina del Carmen Estévez-García, Roberto Belvís, Noemí Morollón, Mónica Cordero-Carcedo, Isabel Mur, Virginia Pomar, Pere Domingo

**Affiliations:** 1 Department of Psychology, Concordia University, Montreal, Canada; 2 Faculty of Health Sciences, Universitat Oberta de Catalunya, Barcelona, Spain; 3 Neuropsychology Unit, Neurology Department, Hospital de la Santa Creu i Sant Pau, Barcelona, Spain; 4 Facultat de Psicologia, Ciències de l’Educació i l’Esport, Blanquerna, Universitat Ramon Llull, Barcelona, Spain; 5 Internal Medicine Department, Hospital de la Santa Creu i Sant Pau, Barcelona, Spain; 6 Neurodiagnostic Department, Hospital de la Santa Creu i Sant Pau, Barcelona, Spain; 7 Neurology Department, Headache Unit, Hospital de la Santa Creu i Sant Pau, Barcelona, Spain; 8 Infectious Disease Unit, Hospital de la Santa Creu i Sant Pau, Barcelona, Spain; The University of New South Wales, Neuroscience Research Australia, AUSTRALIA

## Abstract

Cognitive difficulties are reported as lasting sequelae within post COVID-19 condition. However, the chronicity of these difficulties and related factors of fatigue, mood, and perceived health have yet to be fully determined. To address this, the current longitudinal study aimed to clarify the trends of cognitive test performance and cognitive domain impairment following COVID-19 onset, and whether hospitalization influences outcomes. 57 participants who reported subjective cognitive difficulties after confirmed COVID-19 infection were assessed at baseline (~6 months post COVID-19) and follow-up (~15 months later) visits. Assessments included measures across multiple cognitive domains and self-report questionnaires of fatigue, mood, and overall health. Analyses were conducted in three stages: at the test score level (raw and adjusted scores), at the cognitive domain level, and stratified by hospitalization status during infection. Results at the test-score level indicate that cognitive performance remains relatively stable across assessments at the group level, with no significant improvements in any adjusted test scores at follow-up. Cognitive domain analyses indicate significant reductions in attention and executive functioning impairment, while memory impairment is slower to resolve. On self-report measures, there was a significant improvement in overall health ratings at follow-up. Finally, those hospitalized during infection performed worse on timed cognitive measures across visits and accounted for a larger proportion of cases with short-term and working memory impairment at follow-up. Overall, our findings indicate that cognitive difficulties persist both at test score and cognitive domain levels in many cases of post COVID-19 condition, but evidence suggests some improvement in global measures of attention, executive functioning and overall self-rated health. Furthermore, an effect of hospitalization on cognitive symptoms post COVID-19 may be more discernible over time.

## 1. Introduction

In the years since the initial appearance of COVID-19 on the global stage, we have learned more about its pervasive biological impact during both the acute and post-infection disease stages [1]. With a range of labels applied to long-term effects of this disease across the literature (e.g., Long Covid, post-acute sequelae of COVID-19, post-COVID-19 syndrome; see the World Health Organization’s report [2] for a compiled list of names), the WHO has officially designated the term “post COVID-19 condition” to describe the lasting symptoms of COVID-19 beyond the period of detectable SARS-CoV-2 infection.

Within a constellation of sequelae in post COVID-19 condition, persisting neuropsychiatric and cognitive difficulties have been consistently observed [3]. In a systematic review by Tavares-Júnior and colleagues [4], prevalence of cognitive impairment .ranged from 21% to 65% in samples of previously hospitalized COVID-19 survivors tested 12 or more weeks after infection. Common reports months after contracting COVID-19 include troubles with fatigue, brain fog, and issues with attention and memory processes [5,6]. Comprehensive neuropsychological testing affirms these reports, with cognitive profiles months after disease onset characterized by impaired performance on attentional and executive processing tasks [7–9] and elevated levels of both mental and physical fatigue [10–12] (see Campos et al. [13] for review).

While cognitive impacts of COVID-19 are clearly extending beyond the period of infection, the duration and persistence of these cognitive difficulties in post COVID-19 condition have yet to be fully determined within longitudinal datasets. Baseline/follow-up studies to date have revealed mixed results across various clinical groups. Measured with general cognitive screening tools such as the Montreal Cognitive Assessment (MoCA [14]), a significant number of participants previously hospitalized with COVID-19 showed improvement between 6- and 12-month follow-up assessments, although group median MoCA scores only increased by one point and 44% of participants’ scores still fell in the clinically impaired range [15]. Longitudinal self-report measures in hospitalized patients also reveal subjective reports of improvement in cognitive status but persistent endorsement of memory loss years post hospitalization [16]. Comparing 3- and 12-month follow-up MoCA scores across a range of COVID-19 infection severity groups, researchers found no change in median scores across timepoints but did find a lower percentage of scores (18%) falling in clinical range at follow-up [17]. Overall, these studies provide evidence from screening tools of some improvement, but also indicate lasting cognitive impairment (especially in those who were hospitalized with COVID-19) over 1 year after disease onset.

Beyond screening measures, longitudinal studies with comprehensive neuropsychological assessments have begun to provide nuance with their data, speaking to the specific cognitive domains that are characteristically impacted in post COVID-19 condition. One longitudinal study with previously hospitalized patients observed improvements in the percentage of their sample with impaired attention/processing speed (T1: 40.8%, T2: 28.3%) and long-term verbal memory (T1: 26.3%, T2: 15.1%) between a 5-month post-COVID assessment and 1-year follow-up [18]. Similarly, another longitudinal study found continuing improvements in immediate verbal memory (RAVLT Immediate) and attentional measures (Trail Making Test A) 1 year after disease onset, albeit in a final sample of 16 participants [19]. A third longitudinal study found little change in cognitive status, with comparable levels of impairment (48–56%) at both 3-month and 1-year post-COVID assessments in previously hospitalized patients [20]. Importantly, all articles stress how findings may include some instances of improvement, but they also highlight the persistent nature of COVID-19-associated cognitive difficulties one year (or more) out from infection. Additionally, they highlight an emerging pattern of long-term cognitive difficulties specifically in memory/learning, attention and executive functioning within post COVID-19 condition, although it remains unclear how levels of impairment in these domains change over time.

In sum, the current literature does not offer a clear picture of how cognitive functioning, sufficiently measured to capture distinct cognitive abilities across different domains, changes in this population over time. The present study aims to address this by assessing evolving trends in the long-term clinical profiles of individuals with cognitive complaints post COVID-19 using a longitudinal dataset of comprehensive neuropsychological assessments. Given that our sample included both individuals who were hospitalized during infection and those who were not, we also sought to explore how hospitalization due to COVID-19, a general proxy of disease severity, impacts long-term cognitive profiles. To address these aims, data are analyzed in three stages: (1) at the level of test scores, where measures of cognitive performance, fatigue, depression, anxiety and self-rated health were compared between baseline and follow-up assessments; (2) at the cognitive domain level, where the pervasiveness of cognitive impact at both time points was assessed across various domains of cognitive functioning; and (3) grouped by hospitalization status, where hospitalized versus non-hospitalized participant outcomes were assessed in terms of cognitive tests scores and self-report measures of mood, fatigue, and perceived health, as well as impairment across cognitive domains.

## 2. Methods

### 2.1 Participants

**Fig 1:**
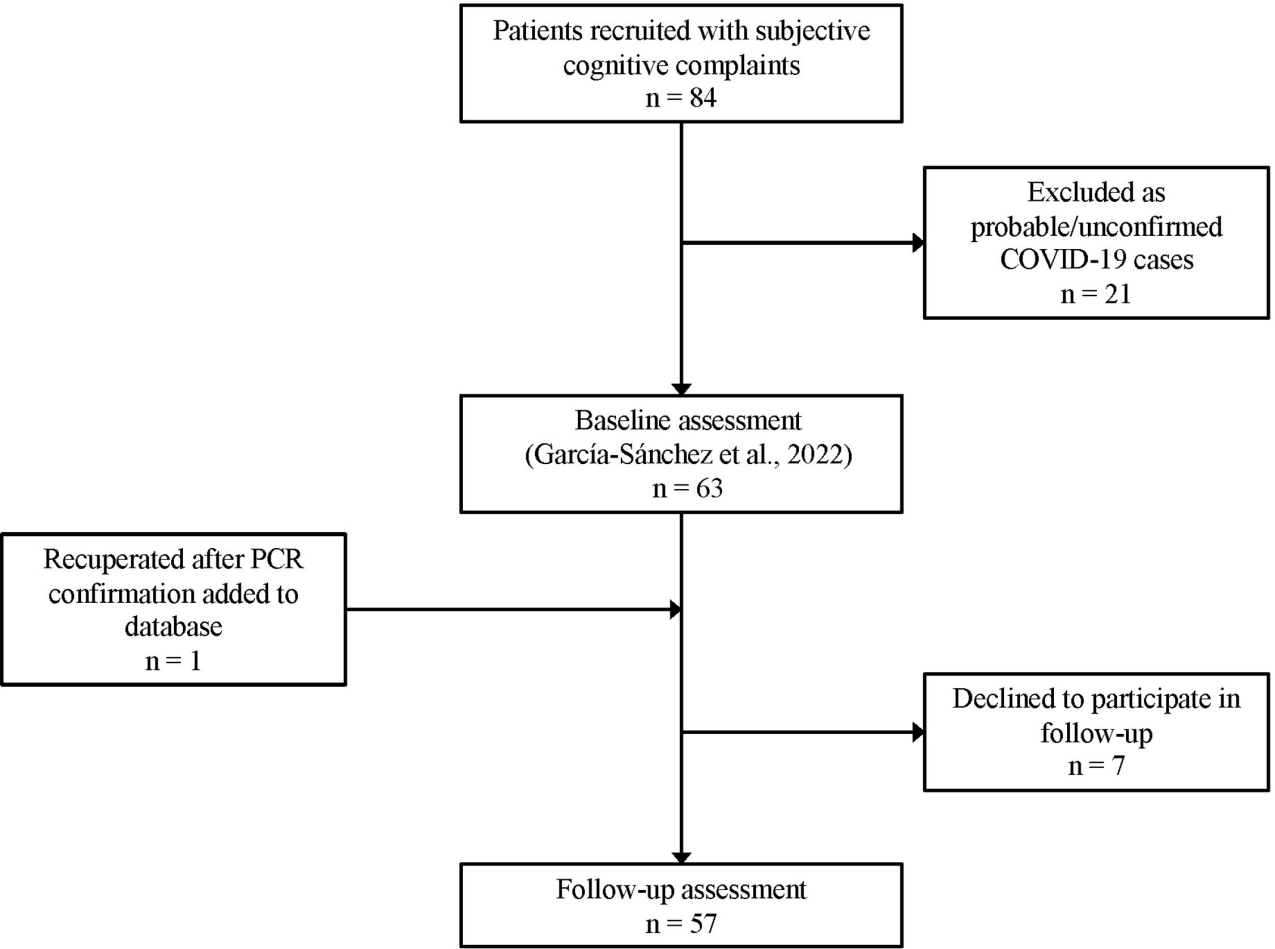
Participant recruitment flow chart for baseline and follow-up studies.

Of the initial 63 subjects included in our baseline study [7], a total of 57 adult participants with post COVID-19 condition completed the follow-up visit (see Fig 1 for recruitment flow chart and Table 1 for total sample characteristics). All participants (1) were symptomatic and tested positive for SARS-CoV-2 via polymerase chain reaction (PCR) and/or serology (anti-SARS-CoV2 IgM or IgG) at the time of infection, (2) reported subjective cognitive complaints following recovery from acute COVID-19 symptoms, (3) were 18 years or older at the time of infection, and (4) contracted COVID-19 prior to availability of vaccines in Spain (i.e., were unvaccinated at the time of infection). Exclusion criteria included documented history of neurological or psychiatric conditions prior to COVID. This study was approved by the Ethics Committee of Hospital de la Santa Creu i Sant Pau (Ref. Nr. HSCSP-20/117) and all participants provided informed written consent.

**Table 1:** Sociodemographic information for total sample and by hospitalization group.

	Total Sample	Status during COVID-19 diagnosis		
		Non-hospitalized	Hospitalized	*p*
**N**	**57**	30	27	
**Sex**				0.012
Females (%)	**37 (65)**	24 (80)	13 (48)	
Males (%)	**20 (35)**	6 (20)	14 (52)	
**Age**				
Mean (SD)	**51.70 (12.80)**	48.63 (12.95)	55.11 (11.96)	0.056
**Education**				
Mean (SD)	**14.34 (3.28)**	14.57 (3.26)	14.08 (3.35)	0.582

*Note*. Reported p-values are derived from a chi-square test for sex and independent samples t-tests for age and education. SD = standard deviation.

Participants were first administered the baseline neuropsychological battery an average of 191.00 days (SD = 99.32), or approximately 6.3 months, after their COVID-19 diagnosis. At that time, participants met the World Health Organization’s definition of *post COVID-19 condition*, with confirmed SARS-CoV-2 infection and clinical symptoms present 3 months or more after the onset of COVID-19 [21]. Follow-up testing occurred an average of 630.28 days (SD = 145.26), or approximately 21 months, post COVID-19 diagnosis. On average, time between evaluations was 439.28 days (SD = 97.50), or approximately 14.6 months. Dates for recruitment via hospital referral services and testing ranged from July 9^th^, 2020 to May 26^th^, 2021 for baseline and July 8^th^, 2021 to December 1^st^, 2022 for follow-up.

### 2.2 Neuropsychological assessment

The follow-up visit consisted of the same comprehensive battery of cognitive measures as administered at baseline visit (see Table 2 for neuropsychological tests and S1 File for test overview and normative data used). All tests were administered in person at baseline and follow-up visits, with the exception of the MoCA at baseline which was administered over the phone. Parallel forms of the MoCA and RAVLT were used at baseline and follow-up assessments to mitigate practice effects.

**Table 2:** Test score distributions across AACN classifications of cognitive performance at baseline and follow-up assessments.

	Baseline				Follow-up				McNemar-Bowker Tests of Symmetry	
	Below average/Exceptionally low (Pc < 8)	Low average (9 ≤ Pc < 24)	Average or above (Pc > 25)	Missing	Below average/Exceptionally low (Pc < 8)	Low average (9 ≤ Pc < 24)	Average or above (Pc > 25)	Missing	χ^2^	*p*-value
**Learning and Long-Term Memory (L+LTM)**										
RAVLT										
Trial 1	10 (17.54)	10 (17.54)	37 (64.91)	–	20 (35.09)	11 (19.3)	26 (45.61)	–	6.095	.107
Trial 5	9 (15.79)	9 (15.79)	39 (68.42)	–	14 (24.56)	8 (14.04)	35 (61.4)	–	1.596	.660
Total	12 (21.05)	16 (28.07)	29 (50.88)	–	20 (35.09)	11 (19.3)	26 (45.61)	–	5.303	.151
Delayed Recall	13 (22.81)	7 (12.28)	37 (64.91)	–	12 (21.05)	7 (12.28)	38 (66.67)	–	0.111	.990
Recognition	13 (22.81)	3 (5.26)	41 (71.93)	–	11 (19.3)	3 (5.26)	43 (75.44)	–	0.286	.897
ROCFT										
Delayed Recall	13 (22.81)	15 (26.32)	29 (50.88)	–	5 (8.77)	14 (24.56)	38 (66.67)	–	5.471	.140
**Visuospatial and Visuoconstructive Abilities (VVA)**										
ROCFT										
Copy Trial	5 (8.77)	14 (24.56)	38 (66.67)	–	11 (19.3)	12 (21.05)	34 (59.65)	–	3.452	.327
Time	5 (8.77)	7 (12.28)	45 (78.95)	–	1 (1.75)	10 (17.54)	46 (80.7)	–	3.077	.380
WAIS-IV										
Block Design	2 (3.51)	8 (14.04)	47 (82.46)	–	3 (5.26)	6 (10.53)	48 (84.21)	–	1.077	.783
**Short-Term and Working Memory (ST/WM)**										
WAIS-IV										
Forward Digit Span	15 (26.32)	6 (10.53)	36 (63.16)	–	11 (19.3)	9 (15.79)	37 (64.91)	–	4.523	.210
Backward Digit Span	6 (10.53)	5 (8.77)	46 (80.7)	–	3 (5.26)	10 (17.54)	44 (77.19)	–	5.571	.134
**Processing Speed (PS)**										
WAIS-IV										
Coding	4 (7.02)	6 (10.53)	47 (82.46)	–	2 (3.51)	9 (15.79)	46 (80.7)	–	1.833	.608
Symbol Search	3 (5.26)	5 (8.77)	49 (85.96)	–	2 (3.51)	5 (8.77)	50 (87.72)	–	0.333	.846
**Language**										
BNT	4 (7.02)	4 (7.02)	49 (85.96)	–	2 (3.51)	4 (7.02)	51 (89.47)	–	0.667	.717
Verbal Fluencies										
Phonemic	9 (15.79)	10 (17.54)	38 (66.67)	–	3 (5.26)	9 (15.79)	45 (78.95)	–	5.886	.117
Semantic	12 (21.05)	6 (10.53)	39 (68.42)	–	10 (17.54)	8 (14.04)	39 (68.42)	–	3.202	.362
**Attention**										
CPT-II										
Omissions %	18 (31.58)	10 (17.54)	29 (50.88)	–	13 (22.81)	10 (17.54)	34 (59.65)	–	2.992	.393
Comissions %	14 (24.56)	13 (22.81)	30 (52.63)	–	15 (26.32)	10 (17.54)	32 (56.14)	–	0.476	.924
Hit RT	23 (40.35)	9 (15.79)	25 (43.86)	–	21 (36.84)	15 (26.32)	21 (36.84)	–	3.067	.381
Hit SE	31 (54.39)	13 (22.81)	13 (22.81)	–	22 (38.6)	20 (35.09)	15 (26.32)	–	7.231	.065
Variability	24 (42.11)	16 (28.07)	17 (29.82)	–	19 (33.33)	23 (40.35)	15 (26.32)	–	3.359	.340
Detectability (d’)	13 (22.81)	23 (40.35)	21 (36.84)	–	10 (17.54)	18 (31.58)	29 (50.88)	–	5.800	.055
Response Style (β)	10 (17.54)	14 (24.56)	33 (57.89)	–	15 (26.32)	14 (24.56)	28 (49.12)	–	2.119	.548
Perseverations %	18 (31.58)	1 (1.75)	38 (66.67)	–	20 (35.09)	1 (1.75)	36 (63.16)	–	0.222	.895
Hit RT Block Change	9 (15.79)	15 (26.32)	33 (57.89)	–	10 (17.54)	15 (26.32)	32 (56.14)	–	2.393	.495
Hit SE Block Change	13 (22.81)	26 (45.61)	18 (31.58)	–	13 (22.81)	20 (35.09)	24 (42.11)	–	4.286	.232
Hit RT ISI Change	16 (28.07)	19 (33.33)	22 (38.6)	–	19 (33.33)	15 (26.32)	23 (40.35)	–	0.895	.827
Hit SE ISI Change	14 (24.56)	16 (28.07)	27 (47.37)	–	16 (28.07)	15 (26.32)	26 (45.61)	–	0.477	.924
**Executive Functioning (EF)**										
Trail Making Test										
A	8 (14.04)	13 (22.81)	36 (63.16)	–	8 (14.04)	7 (12.28)	42 (73.68)	–	6.086	.108
B	10 (17.54)	15 (26.32)	30 (52.63)	2 (3.51)	8 (14.04)	10 (17.54)	38 (66.67)	1 (1.75)	5.655	.130
Stroop Test										
Word Reading	15 (26.32)	14 (24.56)	26 (45.61)	2 (3.51)	17 (29.82)	9 (15.79)	29 (50.88)	2 (3.51)	2.444	.485
Color Naming	16 (28.07)	11 (19.3)	28 (49.12)	2 (3.51)	14 (24.56)	10 (17.54)	31 (54.39)	2 (3.51)	2.300	.513
Inhibition	13 (22.81)	8 (14.04)	34 (59.65)	2 (3.51)	7 (12.28)	13 (22.81)	35 (61.4)	2 (3.51)	3.778	.286

*Note*. Count of participants (percentage of sample) within each AACN performance category reported for each test score. Pc = Percentile. RAVLT = Rey Auditory Verbal Learning Test, ROCFT = Rey-Osterrieth Complex Figure Test, WAIS-IV = Wechsler Adult Intelligence Scale IV, BNT = Boston Naming Test, CPT-II = Conners’ Continuous Performance Test II, RT = Reaction Time, SE = Standard Error, ISI = Inter-Stimulus Interval.

Other clinically relevant factors were also measured at baseline and follow-up: fatigue, measured with the Modified Fatigue Impact Scale (MFIS) [22]; depression and anxiety, measured with the Hospital Anxiety and Depression Scale (HADS) [23]; and self-rated health on a visual analogue scale of current overall health status from the EQ-5D [24].

### 2.3 Analyses

All data entry, inspection, cleaning, and analyses were performed using JASP [25] and the following R packages in RStudio [26]: *tidyverse* [27], *stats* [28], and *statsExpressions* [29]. All data for this study can be found in an anonymized dataset on OSF: https://osf.io/86j3b/.

#### 2.3.1 Test-level analyses

Baseline and follow-up raw scores were obtained from cognitive measures. Age- and education-corrected T-scores and percentiles were then derived using Spanish normative data (see Supporting Information for norms). These adjusted scores were classified into the following clinically relevant categories of performance, following consensus guidelines for labeling cognitive test scores using percentiles (Pc) from the American Academy of Clinical Neuropsychology (AACN) [30]: Below average/Exceptionally low (Pc < 8), Low average (Pc: 9–24), or Average and above (Pc > 25). MoCA scores (version without visual components, max. = 22) were excluded from this classification system, instead using a clinical cut-off score of 18 [31].

Test-level analyses utilized both raw and adjusted test scores. First, we analyzed raw test scores by performing repeated-measures ANCOVAs with Time (baseline vs. follow-up) entered as a within-subjects factor for each raw score on cognitive measures (excluding CPT scores) as well as clinical scores of fatigue, depression and anxiety, and self-rated health. Period since infection (due to varying intervals between infection and assessments), age, education, and sex were controlled for as covariates within these analyses. Missing data was handled using pairwise deletion. Marginal means and test statistics were reported for all significant findings.

Second, we analyzed the distribution of adjusted scores for each cognitive test within the AACN classification system, creating a categorical distribution of scores at baseline and follow-up assessments. McNemar-Bowker tests of symmetry were conducted using proportions of cognitive test scores falling into the three ranges of performance to determine significant changes between the two timepoints. For MoCA scores, proportions of scores falling above and below the cut-off score of 18 at baseline and follow-up were compared.

#### 2.3.2 Domain-level analyses

Cognitive tests at the follow-up study were grouped into domains following the same Principal Components Analysis factors obtained at baseline to aid comparison between timepoints [7]: Learning and Long-term Memory (L+LTM), Visuospatial and Visuoconstructive Abilities (VVA), Short-Term and Working Memory (ST/WM), Processing Speed (PS), Language, Attention, and Executive Functioning (EF). A cognitive domain was considered impaired/affected if it met one of the following conditions: (a) at least 50% of the test scores were labeled as Below average/Exceptionally low; (b) at least 50% of the test scores were Below average/Exceptionally low for tests having single scores; (c) at least 30% of the test scores were Below average/Exceptionally low and 30% of the test scores were labeled as Low average.

To characterize cognitive domain impairment, percentages of affected domains were described at baseline and follow-up. McNemar tests were run to identify significant changes across time between proportions of affected versus non-affected cases in each cognitive domain.

#### 2.3.3 Effect of hospitalization

Analyses examining the effect of hospitalization included mixed ANCOVAs using raw scores, with Time as a within-subjects factor and Hospitalization (hospitalized vs. non-hospitalized) entered as a between-subjects factor. The same covariates (period since infection, age, education, and sex) as previous ANCOVAs were utilized. Statistical techniques comparing a 3 x 3 paired samples design stratified by group are not currently available [32]; consequently, it was not possible to extend McNemar-Bowker tests with adjusted test scores to compare hospitalization status within these analyses.

At the domain level, Pearson’s chi-squared tests of independence were performed for all cognitive domains comparing the frequency of affected domains in hospitalized versus non-hospitalized participants at follow-up assessment.

## 3. Results

### 3.1 Test-level results over time

#### 3.1.1 Raw test scores at baseline and follow-up

Repeated-measures ANCOVAs revealed no statistically significant differences in cognitive performance on neuropsychological measures between assessments (*p* > .050), with the exception of higher scores at follow-up (*M* = 88.494, *SE* = 2.846) compared to baseline (*M* = 88.919, *SE* = 3.530) on Stroop–Word Reading (*F*_(1,49)_ = 4.273, p = .044, η² = .017).

For non-cognitive clinical measures, repeated-measures ANCOVAs did not reveal any statistically significant effects of Time (baseline vs. follow-up) on total fatigue score, anxiety or depression scores (*p* > .050), but there was a significant increase in self-rated health (*F*_(1,51)_ = 5.950, p = .018, η² = .021) from baseline (*M* = 8.665, *SE* = 1.694) to follow-up (*M* = 9.760, *SE* = 1.694).

#### 3.1.2 Adjusted test scores at baseline and follow-up

**Fig 2:**
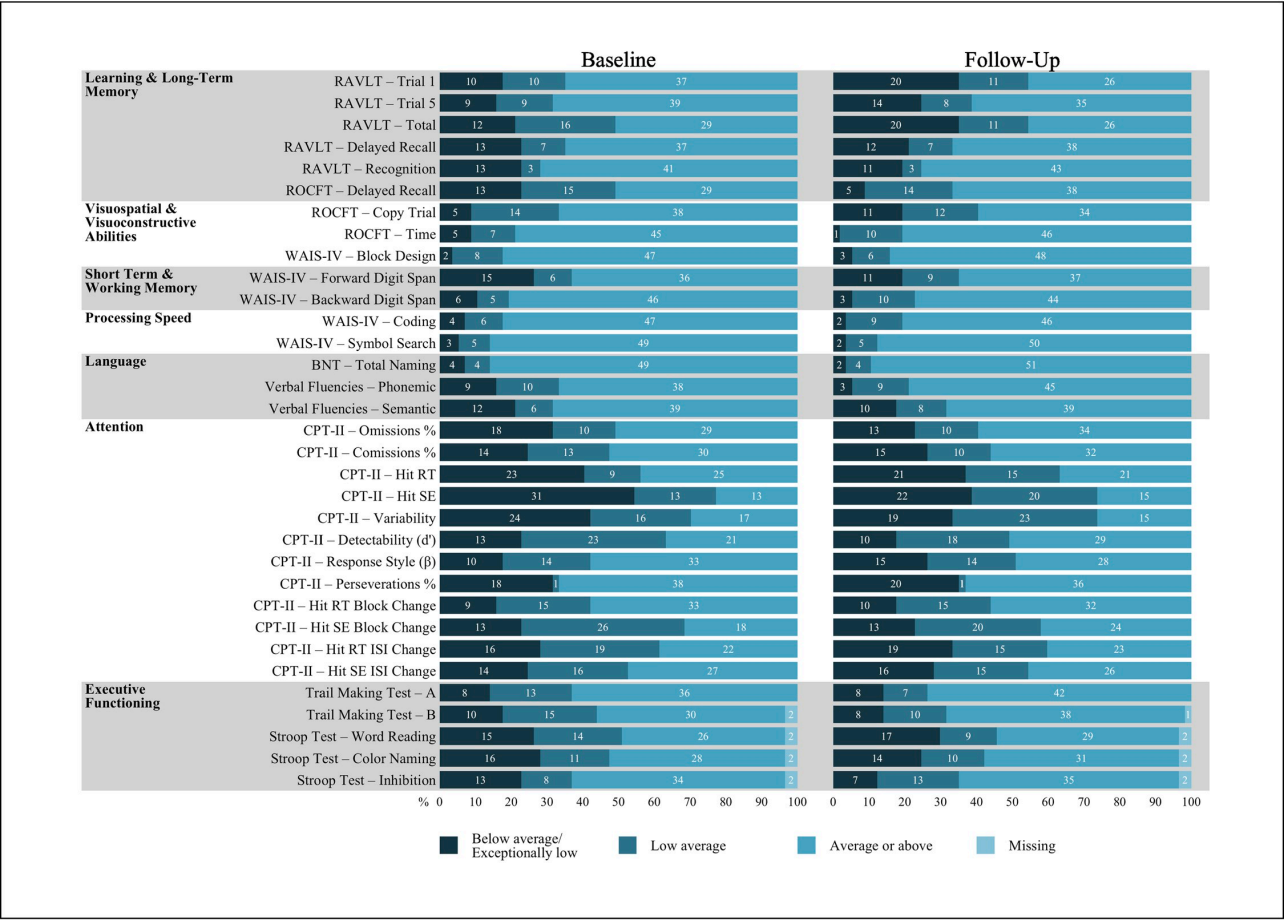
Cognitive test score distributions for baseline and follow-up visits. RAVLT = Rey Auditory Verbal Learning Test, ROCFT = Rey-Osterrieth Complex Figure Test, WAIS-IV = Wechsler Adult Intelligence Scale IV, BNT = Boston Naming Test, CPT-II = Conners’ Continuous Performance Test II, RT = Reaction Time, SE = Standard Error, ISI = Interstimulus Interval.

McNemar-Bowker tests comparing proportions of adjusted scores in AACN categories (Below Average/Exceptionally Low, Low Average, and Average) revealed no statistically significant changes between baseline and follow up (*p* > .050). A McNemar test comparing proportions of MoCA scores falling above and below cut-off also revealed no significant differences between assessment points. See Table 2 for score distributions and test results and Fig 2 for visual distributions of test scores at baseline and follow-up assessments.

### 3.2 Domain-level results over time

While all participants exhibited at least one cognitive domain classified as affected at baseline, 35.09% of participants did not have any affected domains at follow-up. Attention was the most commonly affected cognitive domain at baseline (59.65%) and follow-up (33.33%). This was followed by L+LTM (baseline: 42.11%, follow-up: 31.58%), EF (baseline: 42.11%, follow-up: 21.05%), and ST/WM (baseline: 31.58%, follow-up: 21.05%). The remaining cognitive domains were affected less frequently at follow-up (Language: 10.53%, PS: 3.51%, and VVA: 12.28%). See Table 3 for all percentages at baseline and follow-up.

**Table 3:** Percentages of individuals with affected performances at the domain level.

Domain	Baseline	Follow-Up	
	%	%	*p*-value
Attention	59.65	33.33	0.007*
L+LTM	42.11	31.58	0.201
EF	42.11	21.05	< .001**
ST/WM	31.58	21.05	0.108
Language	17.54	10.52	0.248
VVA	8.77	12.28	0.527
PS	5.26	3.51	0.563
**At least one domain**	**100**	**64.09**	-

*Note*. p-values from McNemar tests demonstrate significant differences between baseline and follow-up in proportion of cases with affected domains. *0.001 ≤ *p* < 0.05 **p < .001. L+LTM = Learning and Long-Term Memory, EF = Executive Function, ST/WM = Short-Term and Working Memory, VVA = Visuospatial and Visuoconstructive Abilities, PS = Processing Speed.

**Fig 3:**
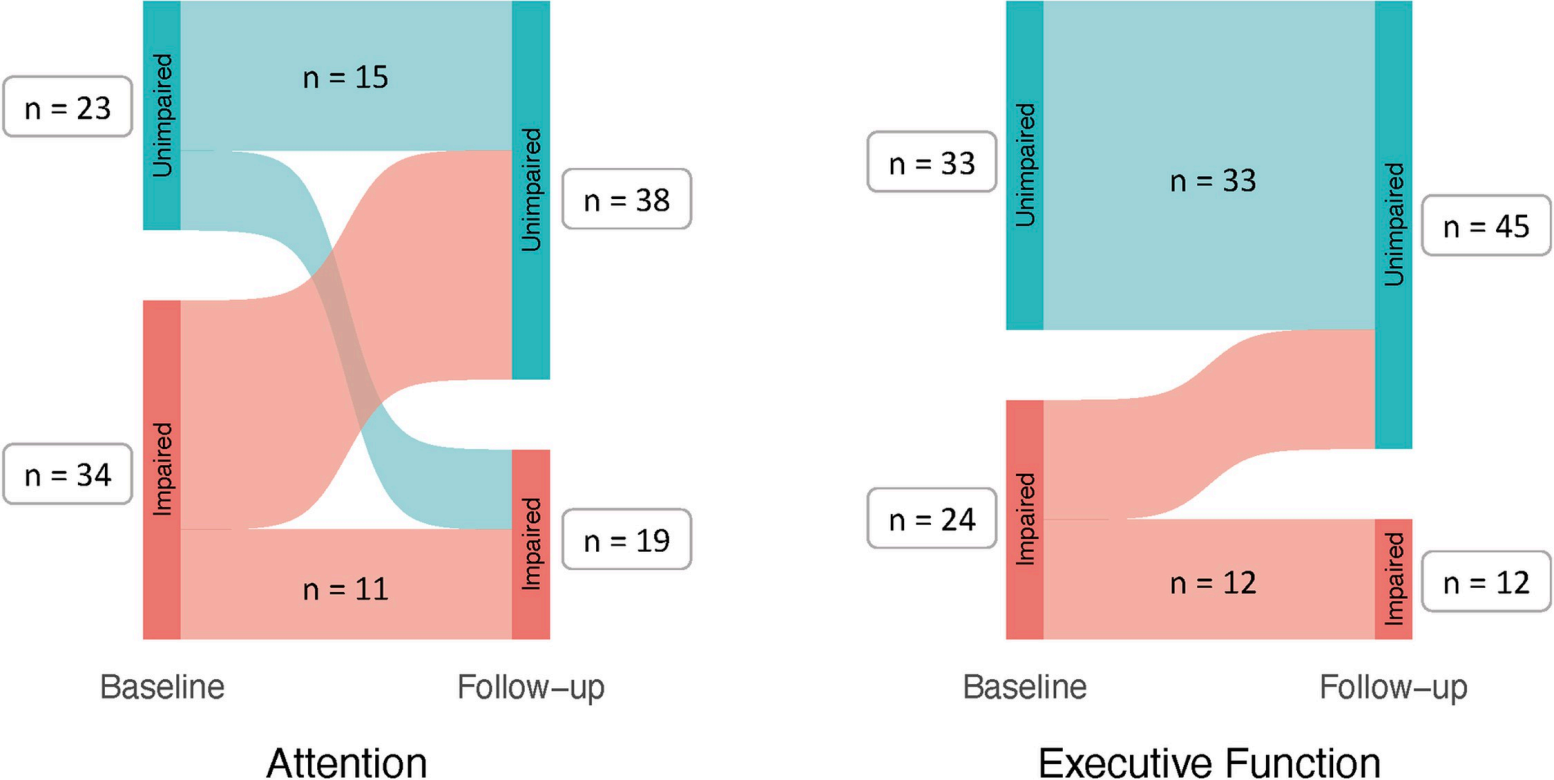
Flow diagrams between baseline and follow-up visits of impaired versus unimpaired cases in attention and executive function domains.

Statistically significant differences in proportions of affected cognitive domains between timepoints were found for Attention (McNemar’s χ^2^ **=** 7.26, *p* = .007, Cohen’s *g* = .24) and EF (McNemar’s χ^2^ **=** 12.00, *p* < .001, Cohen’s *g* = .50). For Attention, 23 of those participants impaired at baseline converted to unimpaired at follow-up and 8 of those unimpaired at baseline were impaired at follow-up. For EF, 12 impaired cases at baseline were unimpaired at follow-up while none of the unimpaired cases became impaired. See Fig 3 for flow diagrams of Attention and EF impairment.

### 3.3 Effects of hospitalization

See Table 1 for sample characteristics by hospitalization group. There was a statistically significant difference in sex between groups, with a higher percentage of women in the non-hospitalized group (80%) than in the hospitalized group (48%; χ^2^ = 6.33, *p* = .012).

At test level, mixed ANCOVAs performed on raw scores revealed no significant effects of Time in cognitive performance between baseline and follow-up assessments, except for Stroop–Word Reading (baseline: *M* = 88.440, *SE* = 2.759; follow-up: *M* = 88.854, *SE* = 3.430; *F*_(1,48)_ = 4.054, p = .050, η² = .017). The increase in self-rated health over time remained statistically significant (baseline: *M* = 8.758, *SE* = 1.696; follow-up: *M* = 9.857, *SE* = 1.696; *F*_(1,50)_ = 5.721, p = .021, η² = .020).

A main effect of Hospitalization in mixed ANCOVAs was revealed for the following cognitive tests: ROCFT–Time (Non-hospitalized: *M* = 134.321, *SE* = 72.481; Hospitalized: *M* = 177.403, *SE* = 71.474; *F*_(1,50)_ = 5.389, p = .024, η² = .060), WAIS–Coding (Non-hospitalized: *M* = 64.254, *SE* = 13.845; Hospitalized: *M* = 52.203, *SE* = 13.652; *F*_(1,50)_ = 11.556, p = .001, η² = .116), WAIS–Symbol Search (Non-hospitalized: *M* = 23.410, *SE* = 7.240; Hospitalized: *M* = 18.148, *SE* = 7.140; *F*_(1,50)_ = 8.057, p = .007, η² = .100), and Stroop–Word Reading (Non-hospitalized: *M* = 94.910, *SE* = 3.861; Hospitalized: *M* = 82.384, *SE* = 3.899; *F*_(1,48)_ = 5.142, p = .028, η² = .069). On all these tests, participants who were hospitalized due to COVID-19 performed poorer than those who were not hospitalized at the time of infection. There was no effect of Hospitalization on clinical measures of fatigue, depression, anxiety or self-rated health.

At the domain level during follow-up, the group of participants with no impaired domains at follow-up was made up of 65% non-hospitalized and 35% previously hospitalized participants. Examining specific domains, hospitalized individuals exhibited a significantly higher proportion of cases with ST/WM impairment compared to non-hospitalized patients (χ^2^ **=** 4.66, *p* = .031, adjusted Cramer’s V = .25), with 50% of those hospitalized demonstrating impairment in short term/working memory versus only 11% of those who were not hospitalized. All chi-squared tests of independence for other cognitive domains revealed no significant proportional differences in impairment between hospitalized and non-hospitalized participants.

## 4. Discussion

The current study examined how cognitive performance and related clinical factors in a group of individuals with cognitive complaints related to post COVID-19 condition evolved over one year between baseline and follow-up neuropsychological assessments. To do so, analyses looked at not only changes in raw test scores, but also changes in scaled test score distributions, changes in impairment at the cognitive domain level, and the effect of hospitalization on long-term recovery.

Overall, our findings suggest that cognitive impairment largely persists one year after COVID-19 infection in individuals with cognitive complaints when assessed using univariate test scores. Test-level analyses reveal very little change in cognitive performance over time when controlling for covariates. Comparing raw scores, only one task of reading speed showed significant change. Given the modest effect size of this difference and our relatively small sample size, this singular difference cannot be interpreted as meaningful change at the test level. Additionally, while there were some shifts in adjusted test score distributions across the two assessments (see Fig 2), none of these changes in proportions were significant.

At the domain level, there was mixed evidence of cognitive change. There was some indication of improvement, with a third of the sample converting from at least one affected domain to no impaired domains. Furthermore, there were significant reductions in proportions of individuals with impairment in Attention and EF domains. In Attention, there were mixed trajectories of participants, with some examples of decline (14.04% of total sample) but an overall group shift towards unimpaired status (40.35% of total sample). In EF, there was a clearer pattern of remission, with half of impaired cases becoming unimpaired (21.05% of total sample) and all previously unimpaired individuals (57.89% of total sample) remaining unimpaired at follow-up.

These patterns of cognitive improvement in domains, albeit mixed, may be reflected in the significant increases in self-rated health observed in our study. Our measure of self-rated health, the visual analogue scale of the EQ-5D, serves as a non-specific measure of quality of life. This scale taps into factors contributing to perceived health beyond the specific symptomology of depression, anxiety, and fatigue, demonstrating only modest correlations with HADS depression and anxiety scores [33]. In fact, this measure is thought to assess aspects of coping such as management of symptoms and psychological disposition beyond that of the HADS [34]. As such, the significant change in this self-rating scale of health is in line with qualitative findings of self-reported improvement in cognitive abilities previously observed in post COVID-19 condition [35]. There has been some debate over how associated subjective reports and objective measures of cognitive impairment are in this population [36,37]. In our sample, subjective improvement in health seem to be mirrored by objective measures when analyzed at a more global domain level and less associated with changes at the test score level. Given this, cognitive functioning measured at the domain level may be more reflective of individuals’ experiences of improvement in cognitive abilities.

However, in conjunction with evidence of improvement, our findings at the domain level also revealed some patterns of lasting cognitive impairment. At follow-up, just over one fifth of our total sample was still impaired in EF and ST/WM, while about one third of the sample was impaired in Attention and L+LTM, at follow-up (see Table 3). This larger picture of some improvement mixed with continued impairment is consistent with previous findings. Comparable studies have reported a common impact in memory, attention, and EF processes, while impairment in language and visuospatial abilities is relatively uncommon [18–20] (for review, see Bertuccelli et al. [3]). Along with some nuanced differences between studies’ findings, the overarching agreement is that these three cognitive processes are the most heavily hit in post COVID-19 condition. Interestingly, while Attention and EF domains may demonstrate partial recovery in our sample, results suggest that proportions of domain-level impairment in memory (L+LTM and ST/WM) remain more stable over time. In line with this, Ferrucci et al. [18] and Diana et al. [19] also found trends of improvement in attention and executive functioning at one year post COVID-19 onset and beyond. While they also found reductions in memory impairment, their combined findings were more ambiguous, with Ferrucci and colleagues reporting improvement in verbal but not visual memory tasks whereas Diana et al.’s findings indicated improvement on verbal learning (not recall) and in long-term visual memory. Our own results, along with those of similar longitudinal neuropsychological studies [18,19], seem to suggest a pattern of partial recovery in attention and executive functioning abilities while recovery of memory processes, both short-term/working and long-term, seems to be less well-defined over time.

Because we did not observe complete remission in cognitive difficulties across our sample, there remains an open question as to what might differentiate individuals who do recover cognitive functioning and those who do not at the time of follow-up assessment. Certainly, there could be a biological (or psychosocial) vulnerability that makes some individuals more susceptible to the long-term effects of the infection and determines their capacity for recovery. Additionally, we might also speculate that the probability of recovery is associated with the patient’s type of neuropsychological profile and underlying neural mechanisms. We observed in our longitudinal study that, overall, there was a substantial decrease in impairments within Attention and EF, while memory impairments were more persistent. Interestingly, in our previous study [7], we found patients’ L+LTM performance was not correlated with their EF and Attention performance. With this finding, we speculated that these two cognitive profiles, one characterized by memory impairment and the other characterized by mixed impairments across Attention and EF domains, were the manifestations of two distinct neurological processes and etiologies, with the former affecting hippocampal structures and the latter impacting more widespread fronto-subcortical networks. As a possible mechanism behind the memory impairment profile, recent studies have provided evidence of a loss of hippocampal neurogenesis in animals and humans infected with SARS-CoV2, albeit in small sample sizes [38]. For cognitive profiles of persisting Attention and EF, generalized neuroinflammation may be a more likely contributor, with another study of post COVID-19 condition finding correlations between executive impairment and immuno-inflammatory markers [39]. Although more research is needed to further substantiate these hypothesized profiles, our findings suggest that these two patterns of impairment could also be prognostic of the long-term trajectory in cognitive recovery.

Hospitalization, a broad proxy for disease severity at the time of infection, appears to have lasting impacts on long-term cognitive performance in post COVID-19 condition. In our sample, scores on multiple timed tests were routinely lower in the hospitalized group compared to the non-hospitalized group, with differences exhibiting medium to large effect sizes (range of η²: .060-.116). Becker et al. [40] found similar results, where hospitalized patients were more likely to be impaired across a variety of cognitive measures. Additionally, the proportion of hospitalized patients with impairment in ST/WM (50%) was significantly higher than the proportion of non-hospitalized participants (13%). This is in line with the findings of Vannorsdall and colleagues [9], who reported more frequent long-term impairment in working memory and executive functioning (measured by oral administration of TMT B, which would have a high loading of working memory given the modality) in ICU patients. Demonstrating a pattern of worse performance on timed tasks and impaired working memory, patients hospitalized with COVID-19 may exhibit a long-term profile of cognitive slowing, requiring more time to complete cognitively demanding tasks. This is consistent with a recent hypothesis of persistent cognitive slowing and hypoarousal as hallmark traits of post COVID-19 condition [41], which may be exacerbated in those who were hospitalized.

Despite this pattern, other cross-sectional studies have found little effect of hospitalization on cognitive performance in the subacute phase of post COVID-19 condition [8,42–44], including our own cross-sectional study where we found hospitalized patients only performed worse on MoCA and WAIS–Coding tests [7]. We also acknowledge that there are many potential mechanisms behind the effect of hospitalization (e.g., psychological stressors such as unemployment or health anxiety) that may not have been captured in our psychological measures (MFIS and HADS). At this point, evidence is mixed as to whether hospitalization during infection is consequential for cognitive function during initial months of recovery. However, as cognitive sequelae evolve over the long term after COVID-19 infection (on average over 21 months in the current study), performance of hospitalization groups may become sufficiently differentiated, with hospitalized patients ultimately demonstrating reliably worse performance on timed tasks and in the working memory domain. Indeed, Fernández-de-las-Peñas et al. [16] found persistent reports of memory difficulties up to 40 months after COVID-19 in hospitalized patients. A review by Ceban et al. [11] found higher proportions of cognitive impairment in hospitalized (30%) versus non-hospitalized (20%) individuals; although this difference did not reach statistical significance, follow-up periods in their meta-analysis ranged from 2.8 to 11.2 months and may not have captured a long-term differentiation between groups. Thus, hospitalization due to COVID-19, and the acute disease severity that it reflects, may become more clearly consequential for cognitive problems in the *years* after disease onset.

In response to the scarcity of studies combining longitudinal methods with comprehensive neuropsychological assessments to examine cognitive functioning in post COVID-19 condition, we believe that a main strength of the current study is the wealth of cognitive data collected and compared over a long-term follow-up design. Furthermore, as our study’s aims were largely exploratory in nature, analyses at both the test and the domain level were conducted to capture cognitive functioning from multiple perspectives. At the test score level, we were able to analyze group differences in raw test scores (ANCOVAs) as well as clinically relevant proportions of test performance (McNemar tests) across time for each cognitive measure. At the domain level, we distilled each individual’s performance across measures of a given domain into an aggregate measure of domain integrity and used this classification to compare the proportions of individuals with impaired domains across time points. While it may initially seem difficult to reconcile largely null findings of cognitive change at the test score level and mixed results of improvement at the domain level, comparisons of more integrated measures of cognitive functioning (i.e., accounting for an individual’s performance across multiple tests within a domain) seemed to be more sensitive to change over time in this subpopulation, in line with their subjective reports of perceived health.

A number of limitations of the current study are also worth noting. First, our study cannot make any conclusive statements regarding the mechanisms underlying observed cognitive change in post COVID-19 condition. This is due to a lack of premorbid measures of cognitive functioning in our sample prior to their COVID-19 infection that prohibits us from making causal claims about the etiology of patients’ deficits. Furthermore, as there were a multitude of potential contributors to cognitive change during and after the pandemic in addition to biological changes peri- and post-infection (e.g., psychological distress, unemployment, health anxiety, etc.), we cannot attribute the lasting cognitive profile in post COVID-19 condition, or the observed effects of hospitalization on speeded tests and ST/WM, solely to effects of the disease itself. Second, this study only consisted of individuals who had already reported subjective cognitive complaints. Although this represents a subpopulation of COVID-19 survivors that is of particular research interest, the propensity to report cognitive complaints may be associated with other personality, psychological (e.g., anxiety), and demographic factors specifically within the post COVID-19 condition population [36]. This might hinder the generalizability of our findings. Finally, our sample size, composed of those participants who returned for a follow-up evaluation after our initial baseline study, was relatively small. This reduced the number of covariates we were able to include in some analyses and may have impacted how reliably we were able to detect meaningful longitudinal changes in this subpopulation.

In conclusion, our results indicate that, in individuals with subjective cognitive complaints post COVID-19, objective cognitive impairment in test scores can linger more than a year past COVID-19 onset. Findings at the cognitive domain level do offer some indication of improvement in attention and executive functioning, with less evidence of change in memory impairment and consistently (low) levels of impairment within other cognitive domains. In parallel, overall participant health ratings show significant improvements over time. Hospitalized patients scored consistently lower than their non-hospitalized counterparts on timed tasks, revealing an effect of hospitalization that may only become significant in the long term (1+ years post COVID-19 onset). Future research should build upon predictive models of long-term cognitive difficulties [45] to clarify what factors shape an individual’s post COVID-19 condition pattern of recovery (e.g., vaccination status, pandemic-related psychological and economic stress, etc.).

## Supporting information

S1 File Neuropsychological tests and normative data. (PDF)
