# Supplementary material for: Evolving trends in neuropsychological profiles of post COVID-19 condition: A 1-year follow-up in individuals with cognitive complaints
Source: PLoS One. 2024 Aug 8;19(8):e0302415. doi: 10.1371/journal.pone.0302415 (PMC11309414; doi:10.1371/journal.pone.0302415)
Supplement: S1 File — (PDF) [file pone.0302415.s001.pdf]

S1 – Neuropsychological Tests and Normative Data (Grunden et al., 2024)

a) *Montreal Cognitive Assessment* [1] (MoCA), a screening tool for cognitive impairment. It includes subtests in attention and concentration, executive functions, memory, language, visuoconstructional skills, conceptual thinking, calculations, and orientation.
b) *Rey's Auditory Verbal Learning Test* [2,3] *(*RAVLT*)*, a test of verbal long-term memory and learning;
c) *Rey-Osterrieth Complex Figure Test* [4–6] (ROCFT), which measures visuoconstructive abilities (copy) and non-verbal long-term memory (delayed recall);
d) *Block Design Test*, a subtest from WAIS-IV [7], which measures visuoconstructive functions;
e) *Digit Span Backward and Digit Span Forward* [7–9], subtests from WAIS-IV, which measure short-term and working memory;
f) *Coding Test*, one of two subtests in the Processing Speed Index from WAIS-IV [7], which measures processing speed by way of visual-motor coordination;
g) *Symbol Search*, the other subtest in the Processing Speed Index from WAIS-IV [7], which measures processing speed in addition to visual discrimination;
h) *Boston Naming Test* [10–12], that assesses picture naming abilities;
i) *Phonetic and semantic fluency tasks* [13,14], which measure verbal fluency;
j) *Conners Continuous Performance Test II* [15] *(*CPT-II*)*, which measures sustained attention.
k) *Trail Making Tests* [8,9,16], which measure the visual attention and processing speed (part A) and task switching (part B);
l) *Stroop task* [17–19], which measures inhibitory control and verbal interference;

1. Nasreddine ZS, Phillips NA, Bédirian V, Charbonneau S, Whitehead V, Collin I, et al. The Montreal Cognitive Assessment, MoCA: A brief screening tool for mild cognitive impairment. J Am Geriatr Soc. 2005;53: 695–699. doi:10.1111/j.1532-5415.2005.53221.x
2. Rey A. L'examen Clinique En Psychologie [The Clinical Psychological Examination] Presses Universitaires de France. Paris, Fr. 1964.
3. Schmidt M. Rey Auditory Verbal Learning Test: RAVLT : a Handbook. Western Psychological Services; 1996.
4. Osterrieth PA. Le test de copie d'une figure complexe; contribution a l'etude de la perception et de la memoire. Arch Psychol (Geneve). 1944.
5. Palomo R, Casals-Coll M, Sánchez-Benavides G, Quintana M, Manero RM, Rognoni T, et al. Estudios normativos españoles en población adulta joven (proyecto NEURONORMA jóvenes): normas para las pruebas Rey-Osterrieth Complex Figure (copia y memoria) y Free and Cued Selective Reminding Test. Neurología. 2012;28: 226–235. doi:10.1016/j.nrl.2012.03.008
6. Pena-Casanova J, Gramunt-Fombuena N, Quinones-Ubeda S, Sanchez-Benavides G, Aguilar M, Badenes D, et al. Spanish Multicenter Normative Studies (NEURONORMA Project): Norms for the Rey-Osterrieth Complex Figure (Copy and Memory), and Free and Cued Selective Reminding Test. Arch Clin Neuropsychol. 2009;24: 371–393. doi:10.1093/arclin/acp041
7. Wechsler D. Escala de Inteligencia Wechsler para adultos IV (Spanish version). Madrid: Pearson; 2012.
8. Tamayo F, Casals-Coll M, Sánchez-Benavides G, Quintana M, Manero RM, Rognoni T, et al. Spanish normative studies in a young adult population (NEURONORMA young adults project): Guidelines for the span verbal, span visuo-spatial, Letter-Number Sequencing, Trail Making Test and Symbol Digit Modalities Test. Neurol (English Ed. 2012;27: 319–329. doi:10.1016/j.nrleng.2012.07.008
9. Peña-Casanova J, Quiñones-Úbeda S, Quintana-Aparicio M, Aguilar M, Badenes D, Molinuevo JL, et al. Spanish multicenter normative studies (NEURONORMA project): Norms for verbal Span, visuospatial Span, letter and number sequencing, trail making test, and symbol digit modalities test. Arch Clin Neuropsychol.

2009;24: 321–341. doi:10.1093/arclin/acp038
10. Goodglass H, Kaplan E, Weintraub S. Boston Naming Test. Lea & Febiger Philadelphia, PA; 1983.
11. Aranciva F, Casals-Coll M, Sánchez-Benavides G, Quintana M, Manero RM, Rognoni T, et al. Spanish normative studies in a young adult population (NEURONORMA young adults project): Norms for the Boston Naming Test and the Token Test. Neurol (English Ed. 2012;27: 394–399. doi:10.1016/j.nrleng.2011.12.010
12. Peña-Casanova J, Quiñones-Úbeda S, Gramunt-Fombuena N, Aguilar M, Casas L, Molinuevo JL, et al. Spanish multicenter normative studies (NEURONORMA project): Norms for boston naming test and token test. Arch Clin Neuropsychol. 2009;24: 343–354. doi:10.1093/arclin/acp039
13. Casals-Coll M, Sánchez-Benavides G, Quintana M, Manero RM, Rognoni T, Calvo L, et al. Spanish normative studies in young adults (NEURONORMA young adults project): Norms for verbal fluency tests. Neurol (English Ed. 2013;28: 33–40. doi:10.1016/j.nrleng.2012.02.003
14. Peña-Casanova J, Quiñones-Úbeda S, Gramunt-Fombuena N, Quintana-Aparicio M, Aguilar M, Badenes D, et al. Spanish multicenter normative studies (NEURONORMA project): Norms for verbal fluency tests. Arch Clin Neuropsychol. 2009;24: 395–411. doi:10.1093/arclin/acp042
15. Conners CK, Staff MHS, Connelly V, Campbell S, MacLean M, Barnes J. Conners' continuous performance Test II (CPT II v. 5). Multi-Health Syst Inc. 2000;29: 175–196.
16. Bowie CR, Harvey PD. Administration and interpretation of the Trail Making Test. Nat Protoc. 2006;1: 2277–2281. doi:10.1038/nprot.2006.390
17. Golden CJ. Stroop. Test Color y Palabras Madrid TEA Ediciones. 1994.
18. Rognoni T, Casals-Coll M, Sánchez-Benavides G, Quintana M, Manero RM, Calvo L, et al. Spanish normative studies in young adults (NEURONORMA young adults project): Norms for Stroop Color–Word Interference and Tower of London-Drexel University tests. Neurol (English Ed. 2013;28: 73–80. doi:10.1016/j.nrleng.2012.02.004

19. Peña-Casanova J, Quiñones-Úbeda S, Gramunt-Fombuena N, Quintana M, Aguilar M, Molinuevo JL, et al. Spanish multicenter normative studies (NEURONORMA project): Norms for the stroop color-word interference test and the tower of London-Drexel. Arch Clin Neuropsychol. 2009;24: 413–429. doi:10.1093/arclin/acp043
